# Astaxanthin alleviates autophagy, inflammation, and oxidative stress in ventilator-associated lung injury rats by inhibiting MAPK/ERK1/2 pathway

**DOI:** 10.1590/acb411726

**Published:** 2026-06-29

**Authors:** Jiayi Zhang, Yifan Wei, Zongyu Chen, Tao Luo, Shiyun Cheng, Jing Wang, Xianming Zhang

**Affiliations:** 1 Affiliated Hospital of Guizhou Medical University – Department of Respiratory and Critical Care Medicine – Yunyan District – China.; 2 Guizhou Medical University – School of Clinical Medicine – Yunyan District – China.; 3 Liupanshui People’s Hospital – Department of Respiratory and Critical Care Medicine – Liupanshui – China.

**Keywords:** Xanthophylls, Ventilator-Induced Lung Injury, MAP Kinase Signaling System, Autophagy

## Abstract

**Purpose:**

Improper use of mechanical ventilation may result in ventilator-induced lung injury (VILI). This work seeks to explore the preventive impact of astaxanthin on VILI.

**Methods:**

In this study, we established the VILI rat and drug intervention model, then evaluated lung tissue damage by observing the appearance and hematoxylin-eosin staining. Oxidative stress was evaluated by determining the levels of myeloperoxidase, catalase, and malondialdehyde.

**Results:**

Our data showed that, compared with the control group, mechanical ventilation increased the mRNA and protein expression levels of interleukin (IL)-1β, IL-6, tumor necrosis factor (TNF)-α, LC3II/I, and Beclin1, as well as the phosphorylation level of ERK1/2 in the lung tissues of VILI rats (*p* < 0.0001), while the expression level of P62 protein decreased (*p* < 0.0001). After pretreatment with astaxanthin, autophagy and inflammatory response were significantly reduced. The phosphorylation of ERK1/2 was also inhibited. Notably, these effects were reversed after using the p-ERK agonist Ro67-7476.

**Conclusion:**

Astaxanthin can inhibit autophagy levels and suppress inflammation and oxidative stress in mechanically ventilated rat lung tissue through inhibiting the MAPK/ERK signaling pathway, thus alleviating lung tissue injury. Therefore, astaxanthin may represent a promising preventive strategy for VILI.

## Introduction

Mechanical ventilation stands as an indispensable therapeutic modality for individuals afflicted with acute lung injury. Nevertheless, it can also precipitate or exacerbate pulmonary damage, a condition termed ventilator-induced lung injury (VILI)^1^. Such patients frequently exhibit multi-organ dysfunction syndrome, with a mortality rate that can reach up to 50%^2^. Hence, there is a pressing need to reveal the underlying mechanisms of VILI and to find novel therapeutic agents for its effective prevention and treatment.

Autophagy is a cellular self-cleaning mechanism that transport damaged organelles and senescent proteins to the lysosome for degradation. Appropriate autophagy plays a beneficial role in cellular homeostasis by reducing excess proteins and organelles. However, excessive autophagy can lead to cell death^3-6^. Research has found that inflammation and autophagy in lung tissue are activated after high tidal volume ventilation. However, inhibiting autophagy can reduce VILI^1,7-9^. The mentioned studies indicated that autophagy is involved in the pathological process of VILI, but its mechanism of occurrence is still unclear.

Astaxanthin, a fat-soluble natural carotenoid initially discovered in deep-sea lobsters, can be extracted from a variety of sources including shrimp, algae, and fish^10-12^. Previous studies have demonstrated that astaxanthin has anti-inflammatory, anti-oxidative stress, and autophagy biological functions^13-17^. Zhang et al.’s study based on acute pancreatitis model showed that astaxanthin could inhibit inflammatory response and autophagy^18^. In addition, Zhu et al., in the mouse model of lipopolysaccharide induced lung injury, also confirmed that astaxanthin can inhibit the inflammatory response^19^. These studies demonstrate that astaxanthin is involved in the repair process of various tissue injuries, but there have been no relevant research reports on whether astaxanthin can play a similar role in VILI.

The extracellular signal-regulated kinase (ERK) 1/2, a member of the MAPK family, plays a key role in regulating inflammatory response and autophagy. Studies have shown that ERK1/2 signaling pathway is involved in the development of VILI^20-23^. In previous studies, the researchers explored astaxanthin’s ability to inhibit inflammation and oxidative stress through the ERK1/2 pathway based on different disease models. Moreover, studies based on spinal cord injury models have shown that astaxanthin can reduce mechanical injury by inhibiting ERK1/2^24^. However, the role and mechanism of astaxanthin in VILI are still unclear. Especially, whether astaxanthin can regulate autophagy through the MAPK/ERK1/2 signaling pathway and participate in the occurrence and development of VILI deserves further investigation.

This study has several limitations that should be acknowledged. First, the mouse model employed may not fully recapitulate the complexity of human VILI. Given this species-specific discrepancy, well-designed clinical studies are urgently needed to clarify the underlying pathogenic mechanisms of human VILI. Second, while lung-protective ventilation strategies with tidal volumes of 6–8 mL/kg have been widely adopted in clinical practice, the current mouse experiment utilized high tidal volume ventilation to induce VILI. Notably, tidal volume magnitude can significantly influence the severity, distribution, and morphological characteristics of lung injury. Thus, the mouse model may not fully mirror the clinical scenario of VILI.

In this study, to investigate whether astaxanthin regulates autophagy in vivo through the MAPK/ERK1/2 signaling pathway to improve VILI, we confirmed the interaction between astaxanthin, autophagy, and VILI by using network pharmacological methods, and MAPK/ERK1/2 may be one of the targets. Subsequently, pretreatment of VILI rats with astaxanthin showed that astaxanthin reduced inflammation and autophagy levels in lung tissue of VILI rats, thereby playing a protective role, while p-ERK agonists reversed the protective effect. Therefore, we speculate that astaxanthin may be a potential strategy for treating VILI.

## Methods

### Reagents and antibodies

Astaxanthin (purity ≥ 97%, synthetic, a racemic mixture of stereoisomers), p-ERK agonist Ro67-7476 (purity ≥ 97%), BCA protein assay kit, RIPA lysis buffer, phenylmethylsulfonyl fluoride (PMSF), phosphatase inhibitors, DAPI, and 5×protein loading buffer were all obtained from Solarbio (Beijing, China). Olive oil was sourced from Shanghai Yuanye (Shanghai, China). TRIzol was obtained from Thermo Fisher Scientific (MA, United States of America). Commercial kits for catalase (CAT), myeloperoxidase (MPO), and malondialdehyde (MDA) were acquired from the Jiancheng Institute of Biotechnology (Nanjing, China). Antibodies against β-actin (20536-1-AP), beclin1 (11306-1-AP), P62 (18420-1-AP), and LC3(14600-1-AP), p-ERK1/2(80031-1-RR), ERK1/2(83533-1-RR), along with the goat-anti-rabbit secondary antibody (SA00001-2), were obtained from Proteintech (Wuhan, China). Antibodies against interleukin (IL)-6 (500286), IL-1β (511369), and tumor necrosis factor-α (TNF-α) (346654) were sourced from ZenBio (Chengdu, China). The goat-anti-rabbit fluorescent secondary antibody (111-165-003) was obtained from Jackson (West Grove, PA, United States of America).

### Network pharmacology

#### Acquisition of astaxanthin targets

Through Pubchem, the linear chemical structure formula SMILES of astaxanthin was obtained from the database, and input the obtained SMILES number into SwissTargetPrediction database predicts the corresponding target genes^25,26^.

### Target acquisition of astaxanthin associated with mechanical ventilation induced-lung injury and autophagy intersection

The Genecards database and Online Mendelian Inheritance in Man (OMIM) platform were accessed^27-29^, using disease and autophagy as keywords, to search the related targets of mechanical ventilation-related lung injury and autophagy. Venny2.1 platform was used to predict the intersection of astaxanthin, mechanical ventilation related lung injury, and autophagy targets.

### Construction of protein interaction network

The intersection targets obtained from item “Acquisition of astaxanthin targets” were imported into the String database^30-34^, the species was selected as “*Homo sapiens*,” the minimum required interaction score was set to 0.900, and the nodes that were not connected to other nodes were removed to obtain the protein-protein interaction network.

### Analysis of target function and pathway annotation

We logged in to the Metascape platform, imported the target gene obtained from item “Construction of protein interaction network”^35^, selected *H. Sapiens* as the species, performed GO functional annotation and KEGG pathway enrichment analysis on it, and used a bioinformatics platform Draw KEGG pathway enrichment bubble chart and GO functional visualization bar chart.

### Animal model and experimental design

The experiment utilized 24 male Sprague Dawley rats obtained from Beijing Vital River Laboratory Animal Technology Co., Ltd (license number: SCXK 2021-0011). Rats aged approximately 6–8 weeks old and weighing 200–260 g were enrolled as experimental subjects in this study. At the time of initial drug administration, the body weight of the rats was approximately 200–220 g, and during mechanical ventilation, their body weight ranged from 240 to 260 g. The rats were acclimatized for three to five days under controlled environment (temperature 20–25°C, humidity 50–70%) in a specific pathogen-free laboratory animal facility in which food and water were available *ad libitum*. Then, the rats were randomly allocated into four groups (n = 6):

- Control group (maintain spontaneous breathing);
- High tidal volume group (VILI);
- High tidal volume+astaxanthin group (VILI+AST, 472-61-7, TargetMol, United States of America);
- High tidal volume+astaxanthin+Ro67-7476group, (VILI+AST+Ro67-7476, p-ERK agonist Ro67-7476, HY-100403, MCE, United States of America).

Both the control group and the VILI group received intragastric administration of olive oil mixed with milk for 14 consecutive days. The other groups were administered astaxanthin orally at the dosage of 60 mg/kg/day^36^, dissolved in olive oil and milk, once daily for 14 days, reaching a final gavage concentration of 6 mg/mL^37,38^. Based on relevant research findings and previous work from our group, six rats were intraperitoneally administered the p-ERK1/2 agonist Ro 67-7476 (dissolved in DMSO, HY-100403, MCE, United States of America) at the dose of 4 mg/kg, 30 minutes prior to mechanical^9^. After a 12-hour fast, the control group underwent tracheal intubation but was not connected to the ventilator, whereas the other groups were weighed, anesthetized, and connected to the animal ventilator, with anesthesia replenished every 40–60 minutes with one-third of the initial dose. Ventilation parameters were set at a tidal volume of 30 mL/kg, respiratory rate of 60 breaths per minute, and positive end-expiratory pressure of 0 cmH_2_O^39^. At the conclusion of the treatment, all animals were euthanized, and lung tissues were obtained for subsequent analysis.

The animal experiment ethics were reviewed and approved by the Animal Experiment Ethics Committee of Guizhou Medical University (approval number: 2101324). All experimental protocols involving rats adhered strictly to the guidelines for the care and use of laboratory animals.

### Processing of lung tissue and collection of bronchoalveolar lavage

Following euthanasia, lung tissue samples were collected from each group, photographed, and compared. They were then rinsed with physiological saline and blotted dry with gauze. The right upper lobe was weighed, dried at 60°C for 72 hours, and reweighed to determine the wet-to-dry weight ratio. Subsequently, the collected bronchoalveolar lavage fluid (BALF) was centrifuged at 4°C and 1,500 rpm for 10 minutes, and the supernatant was collected. Total protein quantification of the BALF was analyzed using a BCA protein assay kit following the manufacturer’s instructions.

### Measurement of myeloperoxidase, catalase, and malondialdehyde

MPO, MDA, and CAT levels in rat lung tissues were determined using commercial kits following the respective protocol steps. The optical density of the specimens was determined using a microplate reader at 460 nm for MPO, 405 nm for CAT, and 532 nm for MDA, respectively, to calculate the concentrations of these enzymes and metabolites.

### Hematoxylin and eosin staining

Lung tissues from each group were fixed, paraffin-embedded, and sectioned. After deparaffinization, rehydration, and hematoxylin and eosin (H&E) staining, the sections were mounted with neutral resin. The morphology of alveoli was observed under optical microscope, and the pathological changes of lung were evaluated. According to the Smith Lung Injury Scoring system, the scoring criteria are:

- There is airway epithelial detachment or alveolar rupture;
- There is edema of the airway epithelium;
- There is infiltration of inflammatory cells in the airway and interstitial tissues;
- There is alveolar atelectasis.

The pathologist used the blind method to score, randomly selected 10 visual fields, and conducted semi-quantitative analysis according to 0~4 points to determine the degree of lung tissue injury:

- 0 = normal lung tissue;
- 1 = minor injury, < 25% of lung injury;
- 2 = moderate injury, 25–50% of lung injury;
- 3 = severe injury, 50–75% lung injury;
- 4 = extremely severe injury, > 75% lung damage.

The final average value is taken as the pathological injury score^40^.

### Immunofluorescence

Immunofluorescence was employed to evaluate the expression of Beclin 1 and LC3 proteins. Paraffin sections (4 μm) were dried at 60°C for 60 minutes, deparaffinized, and hydrated. The slides were immersed in goat serum at 37°C for 0.5 hour to block non-specific binding sites, followed by overnight incubation with primary antibodies against Beclin 1 (1:200) and LC3 (1:200). Subsequently, the sections were warmed to 37°C for 0.5 hour and reacted with fluorescent-labeled secondary antibodies (1:500) in a humidified chamber for 1 hour. After adding DAPI under light-protected conditions, the sections were counterstained for 10 minutes. Finally, the sections were covered and visualized with a fluorescence microscope.

### Reverse transcription-quantitative polymerase chain reaction

Total RNA was isolated from rat lung tissues using TRIzol reagent, and then used to reverse transcribe cDNA with the PrimeScript^TM^ RT reagent (Takara, Dalian, China). The mRNA expression of IL-6, IL-1β, and TNF-α was measured with the TB Green Premix Ex Taq™ II reagent (Takara, Dalian, China) in accordance with the provider’s protocol and standardized to β-actin. All primers were designed and synthesized by Sangon Biotech Co., Ltd (Shanghai, China). The sequences are listed in Table 1. Gene expression was analyzed using the 2^-ΔΔCT^ calculation method^41^.

**Table 1:** Primer Sequences Used for RT-qPCR

Gene	Primer	Sequence (5’-3’)
Beclin1	Forward	GCTTCAAGATCCTGGACCGAGTG
	Reverse	TCCTCTCCTGAGTTAGCCTCTTCC
P62	Forward	GCCTATGGACAAGATGACTGGAATG
	Reverse	TGCTCTCTGTATGCTCCCTTCAC
LC3	Forward	GCAGCAGATCCGTGACC
	Reverse	GCTTCTCACCCTTGTAGCG
IL-6	Forward	CTGGTCTGTTGTGGGTGGT
	Reverse	CTGGTCTGTTGTGGGTGGT
IL-1β	Forward	GACAAGAGCTTCAGGAAGGCAGTG
	Reverse	TCATCATCCCACGAGTCACAGAGG
TNF-α	Forward	CTGTGCCTCAGCCTCTTC
	Reverse	ACTGATGAGAGGGAGCCC
β-actin	Forward	CCCATCTATGAGGGTTACGC
	Reverse	TTTAATGTCACGCACGATTTC

### Western blotting

Pre-chilled RIPA lysis solution, supplemented with PMSF and phosphatase inhibitors, was added to the tissue, which was then minced and thoroughly lysed using an ultrasonic crusher. Subsequently, 5× protein loading buffer was mixed with the samples. Proteins were separated by SDS-PAGE and transferred onto a polyvinylidene fluoride (PVDF) membrane (Millipore). The PVDF membrane was blocked with 5% non-fat milk at 25°C for 1 hour and then incubated with primary antibodies against Beclin1 (1:2,000; Proteintech, China), P62 (1:1,000; Proteintech, China), LC3 (1:1,000; Proteintech, China), ERK (1:2,000; Proteintech, China), p-ERK (1:2,000; Proteintech, China), IL-6 (1:700; Proteintech, China), IL-1β (1:700; Proteintech, China), TNF-α (1:1,000; Proteintech, China), β-actin (1:10,000; Proteintech, China) overnight at 4°C. The membrane was then incubated with a goat anti-rabbit secondary antibody (1:5,000) at 37°C for 1 hour, washed with Tris-buffered saline with Tween-20 (TBST), and developed using enhanced chemiluminescence reagent (ECL, Beyotime, Shanghai, China) on a ChemiDoc Touch Imaging System. Protein expression levels were quantified by ImageJ software (v1.51) and adjusted to β-actin.

### Statistical analysis

Throughout the experiment, related measurements were conducted on lung tissues from different batches of Sprague Dawley rats at least three times. Data are shown as mean ± standard deviation. Statistical analysis and graphing were conducted using GraphPad Prism v8.3. Differences among multiple groups were analyzed using one-way analysis of variance (ANOVA) with subsequent Tukey’s post hoc test. Statistical significance was set at *p* < 0.05. Our experimental results showed robust and statistically significant differences between groups (*p* < 0.01), indicating that the sample size was sufficient to detect meaningful biological effects.

## Results

### Screening and analysis of potential targets of astaxanthin on VILI

**Figure 1:**
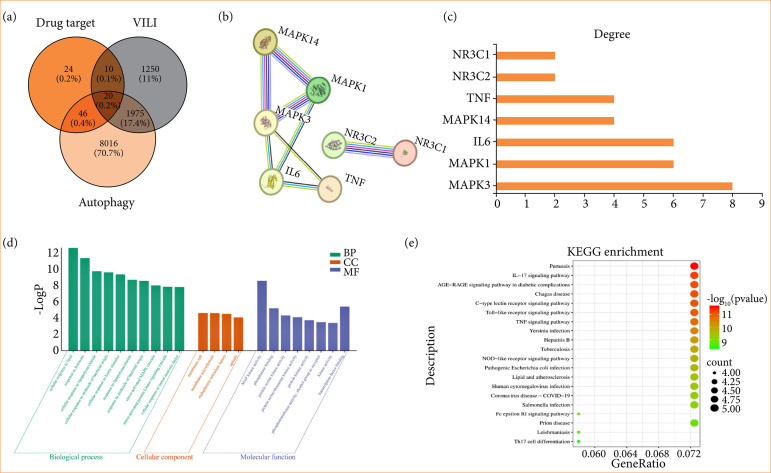
Screening and analysis of potential targets of astaxanthin on ventilator-induced lung injury (VILI). (a) Venn diagram of target interactions between astaxanthin targets and VILI and autophagy, and the numbers of predicted astaxanthin targets. (b) Network diagram of target genes. “Astaxanthin target-autophagy-ventilator-induced lung injury” network, network diagram of 20 intersecting genes. (c) Ranking of degree values of core targets. (d) GO functional visualization bar chart. (e) Enrichment bubble diagram of target genes of astaxanthin.

Source: Elaborated by the authors.

SwissTargetPrediction database could retrieve 100 astaxanthin prediction targets, Genecards database and OMIM database could retrieve 3,255 disease targets related to mechanical ventilation-related lung injury, and 10,057 autophagy related targets. Twenty common targets were obtained by taking the intersection of the three through the Venny2.1 platform (Fig. 1a). The 20 intersection potential targets of astaxanthin in the treatment of mechanical ventilation-related lung injury through autophagy were imported into the String database, and the protein-protein interaction network diagram of astaxanthin in the treatment of mechanical ventilation-related lung injury was obtained (Fig. 1B). After hiding the disconnected nodes in the network, the entire network appeared as seven nodes, including eight edges, with an average node degree value of 0.8. Nodes in the network represent the targets, and edges represent the interaction between the targets. Nodes with more edges indicate that they are more critical in the network, and the interaction of each node is supported by relevant literature evidence. At the same time, key nodes in the network were calculated according to topological parameters, and the degree value was obtained (Fig.1C). The results showed that MAPK3, MAPK1, IL-6, MAPK14, TNF, NR3C2, and NR3C1 astaxanthin acts on the core targets of mechanical ventilation associated lung injury through autophagy.

Subsequently, we performed enrichment analysis of the selected core genes. GO analysis showed that there were 117 biological processes (BP). The data were ranked based on LogP values and the percentage proportion of gene numbers. BP mainly focused on processes such as cellular response to lipid, response to hormone, and cellular response to lipolysis. There are eight molecular functions, mainly involving MAP kinase activity, phosphatase binding, protein serine kinase activity, protein serine/threonine kinase activity, protein kinase activity, and phosphotransferase activity, alcohol groups as acceptors, kinase activity, transcription factor binding, etc. There are four cellular components, mainly composed of membrane raft, membrane microdomain, endothelial lumen, spine, etc. It is suggested that astaxanthin may intervene in the treatment of diseases by participating in lipopolysaccharide, oxidative stress, and other reactions (Fig. 1d). A total of 69 enrichment results were obtained by KEGG Pathway analysis (Fig. 1e). The main pathways involved are Pertussis, IL-17 signaling pathway, AGE-RAGE signaling pathway in diabetic complications, Chagas disease, and C-type lectin receptor signaling pathway, and Toll-like receptor signaling pathway suggests that astaxanthin may regulate and intervene in disease.

### Astaxanthin attenuates lung tissue damage in VILI rats

**Figure 2:**
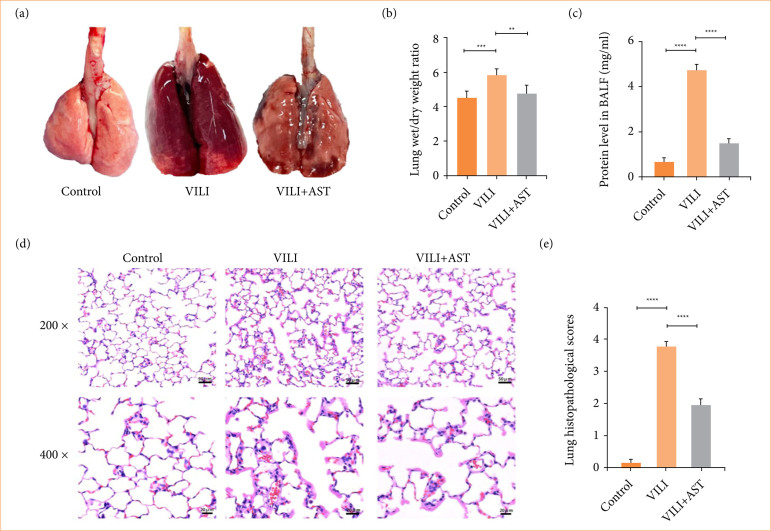
Astaxanthin (AST) attenuates lung tissue damage in ventilator-induced lung injury (VILI) rats. (a) Appearance of lung tissue under the naked eye. (b) Wet/dry weight ratio. (c) Bronchoalveolar lavage fluid (BALF) protein content. (D) Light microscopic field of hematoxylin and eosin stained lung tissue (200× and 400×). (e) Lung histopathological scores.

To examine the effect of astaxanthin in VILI, we induced a rat model of VILI. Macroscopic examination revealed that the lungs of the control group rats appeared pink, whereas those of the rats in the VILI group appeared dark red with obvious congestion and edema. These symptoms were significantly alleviated after pretreatment with astaxanthin (Fig. 2a). Additionally, the wet-to-dry weight ratio and the protein content in BALF were both higher in the VILI group than in the control group, but these indices significantly decreased after astaxanthin treatment (Figs. 2b and 2c). H&E staining images indicated that the lung tissue of rats in the VILI group showed thickened alveolar septa, hemorrhage, and neutrophil infiltration compared to the control group. However, these pathological alterations were markedly improved after pretreatment with astaxanthin (Fig. 2d), and the lung injury scores decreased accordingly (Fig. 2e). In summary, astaxanthin can effectively alleviate lung tissue damage in VILI rats.

**p < 0.01; ***p < 0.001; ****p < 0.0001. Source: Elaborated by the authors.

### Astaxanthin alleviates lung injury in VILI rats by inhibiting inflammation and reducing oxidative stress

**Figure 3:**
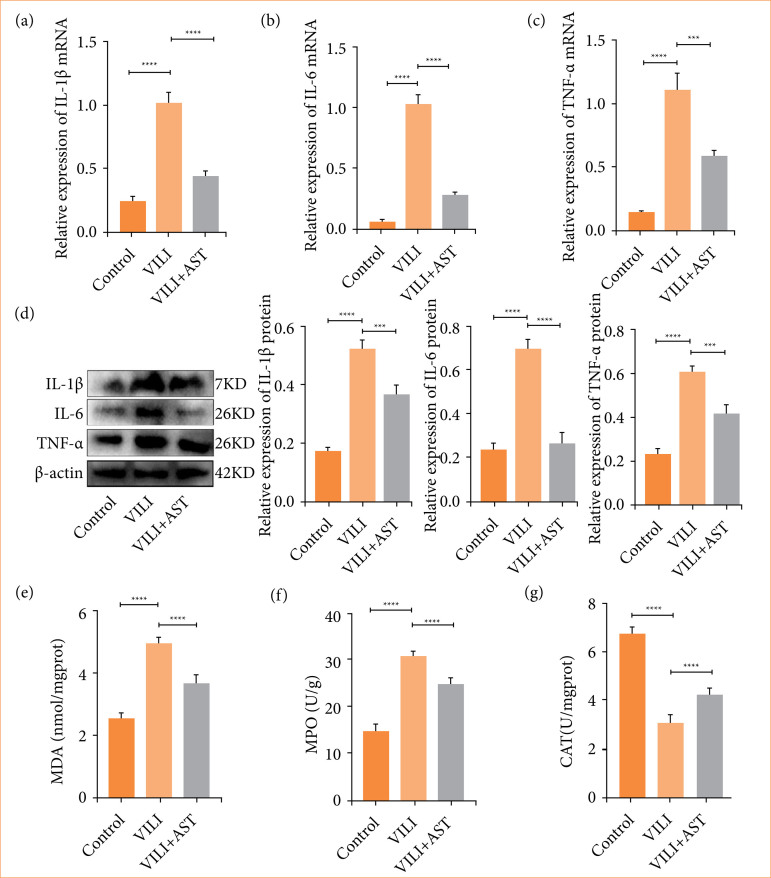
Astaxanthin (AST) alleviates lung injury in ventilator-induced lung injury (VILI) rats by inhibiting inflammation and reducing oxidative stress. (a–d) mRNA expression of interleukin (IL)-1β, IL-6 and tumor necrosis factor- (TNF)-α in rat lung tissues were detected by reverse transcription-quantitative polymerase chain reaction and Western blot, with β-actin as the internal control. (e) Malondialdehyde (MDA), (f) myeloperoxidase (MPO), and (g) catalase (CAT) content in lung tissue were measured by commercial kits.

To elucidate the function of astaxanthin in VILI, we pretreated VILI rats with astaxanthin. We employed reverse transcription-quantitative polymerase chain reaction (RT-qPCR) and Western blotting to detect the mRNA and protein levels of IL-6, IL-1β, and TNF-α. The results showed that both the mRNA and protein expression levels of IL-1β, IL-6, and TNF-α increased significantly compared to the control group. However, after pretreatment with astaxanthin, the expression levels of these factors markedly decreased (Figs. 3a–3d). Furthermore, to determine if there is an imbalance in the oxidation and antioxidant systems in VILI rats and to ascertain the role of astaxanthin, we measured the levels of MDA, MPO, and CAT in the lung tissue. The data demonstrated that compared with the control group, the levels of MDA and MPO were notably increased, while the level of CAT was markedly decreased in the VILI group. Pre-administration with astaxanthin significantly reduced the levels of MDA and MPO and increased the level of CAT (Figs. 3e and 3f). The results suggest that astaxanthin can inhibit inflammatory response and reduce oxidative stress in lung tissue caused by mechanical ventilation, thereby exerting a protective effect on the lungs.

***p < 0.001; ****p < 0.0001. Source: Elaborated by the authors.

### Astaxanthin alleviates lung injury in VILI rats by inhibiting autophagy

To investigate the effect of astaxanthin on alleviating lung injury in VILI rats by inhibiting autophagy, we conducted experiments by establishing a VILI rat model and treating with astaxanthin to detect autophagy indicators. In VILI model rats, the upregulation of autophagy markers (LC3II/I, Beclin1) was accompanied by parallel increases in ERK1/2 phosphorylation. Western blotting and RT-qPCR showed that compared to the control group, the protein expression levels of P62 protein decreased in the VILI group. Following astaxanthin pretreatment, the downregulation of autophagy markers was consistent with changes in ERK1/2 phosphorylation levels, and the expression level of P62 protein increased relatively.

**Figure 4:**
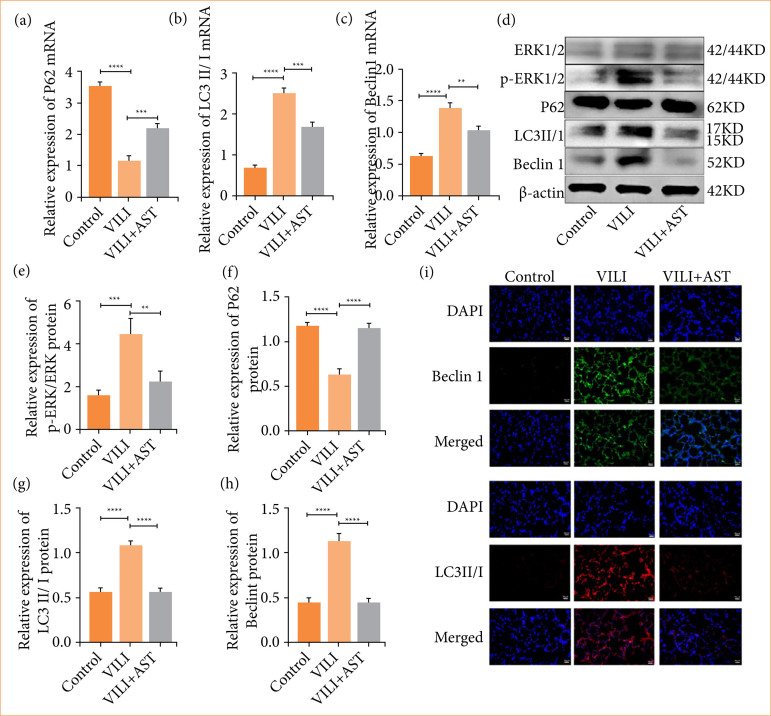
Astaxanthin (AST) alleviates lung injury in ventilator-induced lung injury (VILI) rats by inhibiting autophagy. (a–c) mRNA expression levels of autophagy markers Beclin1, LC3 II/I, and P62 were measured by reverse transcription-quantitative polymerase chain reaction, with β-actin as the internal control. (d–h) Protein expression levels of Beclin1, LC3 II/I, P62, ERK1/2 and p-ERK1/2 were measured by Western blotting, with β-actin as the internal control. (i) Representative images of immunofluorescence for Beclin1 and LC3 II/I.

Similarly, the results also confirmed the activation of ERK1/2 phosphorylation in VILI, and astaxanthin can further inhibit ERK1/2 phosphorylation, but it does not affect the expression of total ERK1/2 protein (Figs. 4a–4h). Immunofluorescence detection also demonstrated that the protein expression levels of Beclin1 and LC3II/I were significantly elevated in the VILI group compared to the control group. After astaxanthin treatment, the protein expression levels of both markers decreased (Fig. 4i). These results indicate that astaxanthin can inhibit autophagy of lung tissues of VILI rats, thereby alleviating lung injury in VILI rats.

**p < 0.01; ***p < 0.001; ****p < 0.0001. Source: Elaborated by the authors.

### Astaxanthin alleviates VILI by reducing inflammation and oxidative stress through the ERK1/2 pathway

**Figure 5:**
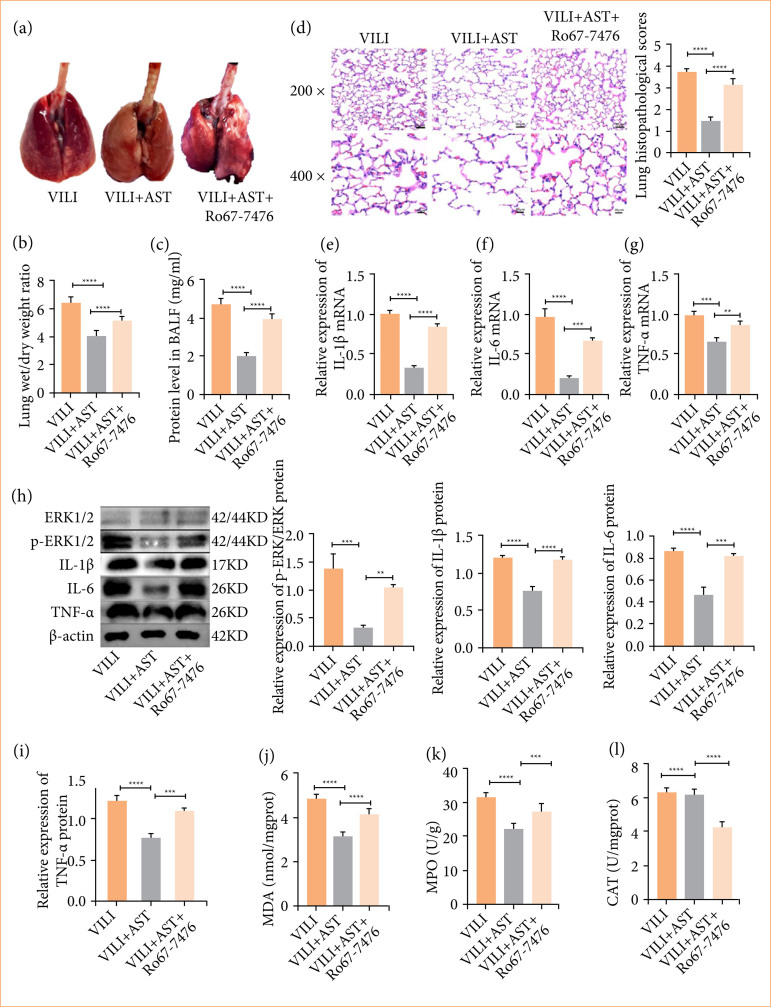
Astaxanthin (AST) alleviates ventilator-induced lung injury (VILI) by reducing inflammation and oxidative stress through the ERK1/2 pathway. (a) Appearance of lung tissue under the naked eye. (b) Wet/dry weight ratio. (c) Bronchoalveolar lavage fluid (BALF) protein content. (d) Light microscopic field of hematoxylin and eosin-stained lung tissue (200× and 400×). Lung histopathological scores. (e–g) mRNA expression of interleukin (IL)-1β, IL-6, and tumor necrosis factor- (TNF)-α in rat lung tissues were detected by reverse transcription-quantitative polymerase chain reaction. (h andi) Protein expression of IL-1β, IL-6, and TNF-α and ERK1/2, p-ERK1/2 in rat lung tissues were detected by Western blot, with β-actin as the internal control. Protein expression of (j) malondialdehyde (MDA), (k) myeloperoxidase (MPO), and (l) catalase (CAT) content in lung tissue were measured by commercial kits.

To verify whether astaxanthin can alleviate mechanical ventilation induced lung injury in rats by regulating the MAPK/ERK1/2 pathway, we pretreated VILI rats with astaxanthin and p-ERK agonist Ro67-7476. Compared with the HV group, astaxanthin can reduce the hyperemia, bleeding spots and edema outside lung tissue (Fig. 5a). The wet-to-dry weight ratio and BALF protein concentration in lung tissue were decreased (Figs. 5b and 5c). H&E staining showed a decrease in neutrophil infiltration and bleeding in the lung interstitium, and a reduction in pathological damage to the lung tissue (Fig. 5d). Surprisingly, Ro67-7476 reversed the protective effect of astaxanthin on lung tissue damage.

**p < 0.01; ***p < 0.001; ****p < 0.0001. Source: Elaborated by the authors

Meanwhile, the results also showed that compared with the VILI group, astaxanthin can reduce MV-induced high expression of the pro-inflammatory factor IL-1β, IL-6 and TNF-α, both at mRNA and protein levels. However, the application of Ro67-7476 reversed the protective effect of astaxanthin. In addition, the expression of p-ERK1/2 was down-regulated after intervision with astaxanthin. Conversely, the reversal effect of astaxanthin was decreased by the application of Ro67-7476 (Figs. 5e–5i). Moreover, the levels of MDA and MPO were relatively decreased, while the level of CAT was relatively increased in the VILI+AST group. Similarly, the protective effect of astaxanthin on oxidative stress in lung tissue has been reversed by p-ERK agonist Ro67-7476 (Figs. 5j–5l). In summary, these results indicate that astaxanthin can alleviate VILI by inhibiting ERK1/2 phosphorylation.

### Astaxanthin alleviates VILI by reducing autophagy through the ERK1/2 pathway

**Figure 6:**
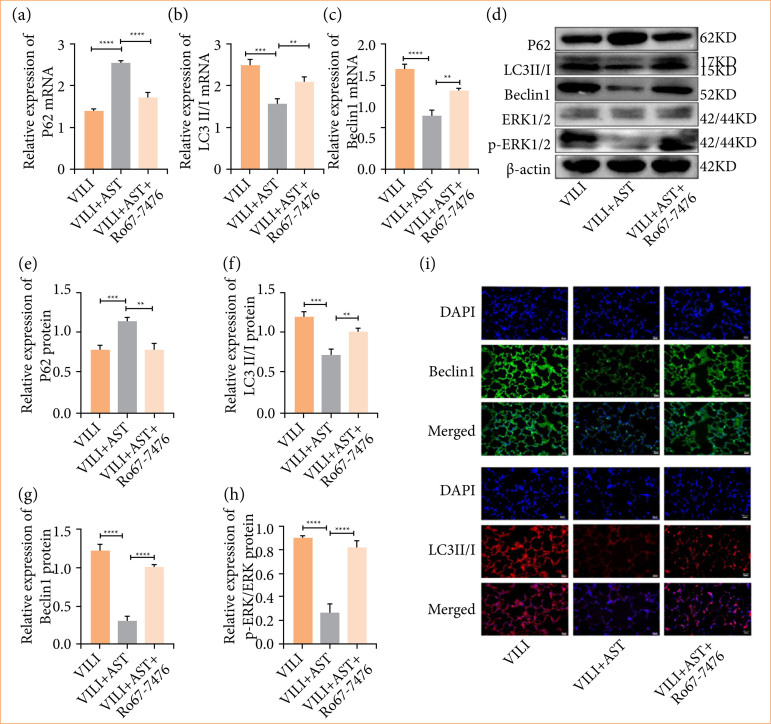
Astaxanthin (AST) alleviates ventilator-induced lung injury (VILI) by reducing autophagy through the ERK1/2 pathway. (a–c) mRNA expression levels of autophagy markers Beclin1, LC3 II/I, and P62 were measured by reverse transcription-quantitative polymerase chain reaction, with β-actin as the internal control. (d–h) Protein expression levels of autophagy markers Beclin1, LC3 II/I, P62 and ERK1/2, p-ERK1/2 were measured by Western blotting, with β-actin as the internal control. (i) Representative images of immunofluorescence for beclin1 and LC3 II/I.

To demonstrate that astaxanthin can decrease autophagy levels by inhibiting ERK1/2 phosphorylation, thereby alleviating lung tissue damage in VILI rats, we used RT-qPCR and Western blotting to detect the mRNA and protein levels of autophagy-related marker Beclin1, LC3II/I, and P62.The results showed that the mRNA and protein levels of Beclin1 and LC3II/I in the VILI+AST group were significantly lower than those in the VILI group, while the expression level of P62 significantly elevated. However, p-ERK agonist Ro67-7476 intervention reversed the beneficial effects of astaxanthin (Figs. 6a–6h). In addition, immunofluorescence detection also demonstrated that the protein expression levels of Beclin1 and LC3II/I were significantly reduced in the VILI+AST group compared to the VILI group, but these changes were reversed following p-ERK agonist Ro67-7476 intervention (Fig. 6i). Thus, it is concluded that astaxanthin can reduce autophagy levels by inhibiting ERK1/2 phosphorylation, thereby exerting a protective effect in VILI rats lung tissue.

**p < 0.01; ***p < 0.001; ****p < 0.0001. Source: Elaborated by the authors.

## Discussion

Mechanical ventilation is a critical life-support measure for patients with acute respiratory distress syndrome, but it can also induce or exacerbate lung tissue damage, leading to VILI^42-45^. Therefore, identifying new drugs or techniques for VILI prevention and treatment holds significant clinical value. The present study suggested that astaxanthin can alleviate VILI by inhibiting ERK/1/2 phosphorylation, reducing the release of inflammatory mediators, oxidative stress, and autophagy levels.

Astaxanthin is a potent antioxidant widely present in nature. Its potential antioxidant and anti-inflammatory effects have shown application potential in various clinical conditions, including diabetes, neurodegenerative disorders, gastrointestinal diseases, hepatic and renal diseases, tumors, and skin repair^46-48^. Previous studies have found that astaxanthin can participate in regulating autophagy levels and alleviating tissue damage^18,49-52^. In our study, astaxanthin can significantly decreased the levels of pro-inflammatory factors IL-6, IL-1β, TNF-α and autophagy related markers Beclin1 and LC3II/I in VILI rats, while increased the expression of P62. Meanwhile, enzyme indexes MDA and MPO related to oxidative damage were significantly reduced, and antioxidant indexes CAT were significantly increased. The above results confirmed that astaxanthin can exert protective effects in VILI lung tissue through anti-inflammatory, antioxidant, and autophagic inhibition. However, the mechanism by which astaxanthin regulates inflammatory, oxidative stress, and autophagy has not been cleared in VILI.

In the further mechanism study, we firstly based on the network pharmacological findings that the key intersecting targets of astaxanthin, VILI, and autophagy include MAPK1, MAPK3, MAPK14, NR3C2 NR3C1, TNF, IL-6, and other 20 genes. Among them, the MAPK family members rank among the top. Previous studies have found that astaxanthin can exert beneficial effects by inhibiting the phosphorylation of ERK, thereby reducing autophagy levels^52^. Our study further confirmed that MV can significantly induce the phosphorylation of ERK1/2, and astaxanthin can reverse the activation of ERK1/2 by MV.

In addition, to further elucidate whether astaxanthin reduces inflammation, oxidative stress, and autophagy levels in VILI dependent on the MAPK/ERK signaling pathway, we introduced p-ERK agonist Ro 67-7476 into the existing astaxanthin pretreatment regimen. Our research data showed that, with the application of Ro67-7476, the expression of the pro-inflammatory factors IL-1β, IL-6, TNF-α and autophagy related markers Beclin1 and LC3II/I were increased relatively in VILI rats, while reduced the expression of P62. On the other hand, the levels of MDA and MPO were relatively elevated, while the level of CAT was relatively decreased, the p-ERK agonist Ro67-7476 reversed the protective effect of astaxanthin. Taken together, these results suggest that astaxanthin reduces inflammation, oxidative stress, and autophagy levels by inhibiting the MAPK/ERK1/2 pathway, thereby alleviating lung tissue damage in VILI rats.

**Figure 7:**
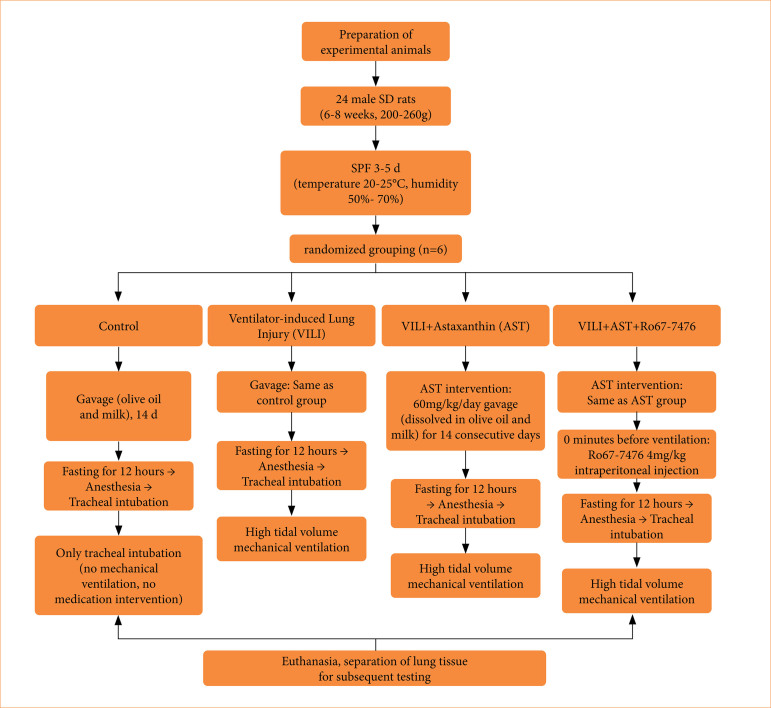
Schematic diagram of the overall research strategy for astaxanthin in treating ventilator-induced lung injury (VILI).

SD: Sprague Dawley; SPF: specific pathogen free. Source: Elaborated by the authors.

In short, our experimental results demonstrate that astaxanthin can reduce inflammation, oxidative stress, and autophagy levels by inhibiting the MAPK/ERK1/2 pathway, thereby alleviating lung tissue damage in VILI rats (Fig. 7). However, due to experimental limitations, further *in-vitro* cell experiments were not conducted in this study, and the protective effect of astaxanthin on VILI has not been validated in clinical prevention and treatment. These two limitations need to be addressed in future research.

### Limitations

Our study has a notable limitation in the lack of direct *in-vivo* functional lung measurements, such as lung compliance, airway resistance, or arterial blood gas analysis. While our data robustly demonstrate that AST treatment alleviates histological injury, inflammation, and oxidative stress, we did not quantitatively assess how these improvements translate into real-time pulmonary mechanical function or gas exchange efficiency. This omission was primarily due to the technical focus and scope of the present study, which was designed to elucidate the underlying molecular mechanisms (*e.g.*, MAPK/ERK1/2 pathway). Nevertheless, the significant reduction in key pathological hallmarks—including alveolar wall thickness, inflammatory cell infiltration, and the levels of pro-inflammatory cytokines—strongly suggests a concomitant improvement in overall lung function. Future studies will directly address this by incorporating techniques such as invasive plethysmography to obtain precise measurements of lung mechanics, thereby providing a direct functional link to the molecular pathways identified here.

## Conclusion

Our findings suggest that astaxanthin may inhibit the MAPK/ERK1/2 signaling pathway, thereby attenuating lung tissue injury in rats with VILI. Mechanistically, astaxanthin suppresses autophagy, inflammation, and oxidative stress in lung tissues of mechanically ventilated rats by blocking the MAPK/ERK signaling pathway. Therefore, astaxanthin may serve as a promising preventive strategy for VILI.

## Data Availability

The datasets generated and analyzed during the study are available from the corresponding author upon reasonable request.

## References

[1] Ge X, Sun J, Fei A, Gao C, Pan S, Wu Z (2019). Hydrogen sulfide treatment alleviated ventilator-induced lung injury through regulation of autophagy and endoplasmic reticulum stress. Int J Biol Sci.

[2] Vieillard-Baron A, Matthay M, Teboul JL, Bein T, Schultz M, Magder S, Marini JJ (2016). Experts’ opinion on management of hemodynamics in ARDS patients: focus on the effects of mechanical ventilation. Intensive Care Med.

[3] Deretic V (2011). Autophagy in immunity and cell-autonomous defense against intracellular microbes. Immunol Rev.

[4] Preetha Rani, Salin Raj, Nair A, Ranjith S, Rajankutty K, Raghu KG (2022). *In vitro* and *in vivo* studies reveal the beneficial effects of chlorogenic acid against ER stress mediated ER-phagy and associated apoptosis in the heart of diabetic rat. Chem Biol Interact.

[5] Kabat AM, Pott J, Maloy KJ (2016). The mucosal immune system and its regulation by autophagy. Front Immunol.

[6] Liang J, Zhang H, Zeng Z, Wu L, Zhang Y, Guo Y, Lv J, Wang C, Fan J, Chen N (2021). Lifelong aerobic exercise alleviates sarcopenia by activating autophagy and inhibiting protein degradation via the AMPK/PGC-1α signaling pathway. Metabolites.

[7] Nosaka N, Martinon D, Moreira D, Crother TR, Arditi M, Shimada K (2020). Autophagy protects against developing increased lung permeability and hypoxemia by down regulating inflammasome activity and IL-1β in LPS plus mechanical ventilation-induced acute lung injury. Front Immunol.

[8] Wang Z, Chen M, Pan X, Wang L, Yin C, Lin Q, Jiang J, Zhang Y, Wan B (2022). Knockout of GGPPS1 restrains rab37-mediated autophagy in response to ventilator-induced lung injury. Hum Cell.

[9] Zhu Q, Wu K, Lv J, Yang R, Li C, Liu W, Zhang J, Lian S, Wang L, Zhang X (2025). CAVIN2 attenuates ventilator-induced lung injury in rats by MAPK/ERK1/2 signaling pathway. Int Immunopharmacol.

[10] Gupta A, Barrow CJ, Puri M (2022). Multiproduct biorefinery from marine thraustochytrids towards a circular bioeconomy. Trends Biotechnol.

[11] Bi F, Xiang H, Li J, Sun J, Wang N, Gao W, Sun M, Huan Y (2023). Astaxanthin enhances the development of bovine cloned embryos by inhibiting apoptosis and improving DNA methylation reprogramming of pluripotency genes. Theriogenology.

[12] Jabarpour M, Aleyasin A, Nashtaei MS, Lotfi S, Amidi F (2023). Astaxanthin treatment ameliorates ER stress in polycystic ovary syndrome patients: a randomized clinical trial. Sci Rep.

[13] Faraone I, Sinisgalli C, Ostuni A, Armentano MF, Carmosino M, Milella L, Russo D, Labanca F, Khan H (2020). Astaxanthin anticancer effects are mediated through multiple molecular mechanisms: A systematic review. Pharmacol Res.

[14] Alugoju P, Krishna Swamy VKD, Anthikapalli NVA, Tencomnao T (2023). Health benefits of astaxanthin against age-related diseases of multiple organs: A comprehensive review. Crit Rev Food Sci Nutr.

[15] Ma H, Chen S, Xiong H, Wang M, Hang W, Zhu X, Zheng Y, Ge B, Li R, Cui H (2020). Astaxanthin from Haematococcus pluvialis ameliorates the chemotherapeutic drug (doxorubicin) induced liver injury through the Keap1/Nrf2/HO-1 pathway in mice. Food Funct.

[16] Kato T, Kasai T, Sato A, Ishiwata S, Yatsu S, Matsumoto H, Shitara J, Murata A, Shimizu M, Suda S, Hiki M, Naito R, Daida H (2020). Effects of 3-month astaxanthin supplementation on cardiac function in heart failure patients with left ventricular systolic dysfunction-a pilot study. Nutrients.

[17] Chang CH, Chen KC, Liaw KC, Peng CC, Peng RY (2020). Astaxanthin protects PC12 cells against homocysteine- and glutamate-induced neurotoxicity. Molecules.

[18] Zhang H, Yang W, Li Y, Hu L, Dai Y, Chen J, Xu S, Xu X, Jiang H (2018). Astaxanthin ameliorates cerulein-induced acute pancreatitis in mice. Int Immunopharmacol.

[19] Zhu L, Wu H, Ma Z, Dong D, Yang Z, Tian J (2022). Astaxanthin ameliorates lipopolysaccharide-induced acute lung injury via inhibition of inflammatory reactions and modulation of the SOCS3/JAK2/STAT3 signaling pathways in mice. Food Funct.

[20] Ren R, Ruan Z, Ding H, Du J, Yu W (2020). Phosphoproteome profiling provides insight into the mechanisms of ventilator-induced lung injury. Exp Ther Med.

[21] Lionetti V, Lisi A, Patrucco E, De Giuli P, Milazzo MG, Ceci S, Wymann M, Lena A, Gremigni V, Fanelli V, Hirsch E, Ranieri VM (2006). Lack of phosphoinositide 3-kinase-gamma attenuates ventilator-induced lung injury. Crit Care Med.

[22] Zhu CH, Yu J, Wang BQ, Nie Y, Wang L, Shan SQ (2020). Dexmedetomidine reduces ventilator-induced lung injury via ERK1/2 pathway activation. Mol Med Rep.

[23] He S, Chen Z, Xue C, Zhou L, Li C, Jiang W, Lian S, Shen Y, Liao M, Zhang X (2022). MiR-9a-5p alleviates ventilator-induced lung injury in rats by inhibiting the activation of the MAPK signaling pathway via CXCR4 expression downregulation. Int Immunopharmacol.

[24] Fakhri S, Dargahi L, Abbaszadeh F, Jorjani M (2019). Effects of astaxanthin on sensory-motor function in a compression model of spinal cord injury: Involvement of ERK and AKT signalling pathway. Eur J Pain.

[25] Daina A, Michielin O, Zoete V (2019). SwissTargetPrediction: updated data and new features for efficient prediction of protein targets of small molecules. Nucleic Acids Res.

[26] Gfeller D, Grosdidier A, Wirth M, Daina A, Michielin O, Zoete V (2014). SwissTargetPrediction: a web server for target prediction of bioactive small molecules. Nucleic Acids Res.

[27] Rebhan M, Chalifa-Caspi V, Prilusky J, Lancet D (1997). GeneCards: integrating information about genes, proteins and diseases. Trends Genet.

[28] Amberger JS, Bocchini CA, Schiettecatte F, Scott AF, Hamosh A (2015). OMIM.org: Online Mendelian Inheritance in Man (OMIM®), an online catalog of human genes and genetic disorders. Nucleic Acids Res.

[29] Safran M, Dalah I, Alexander J, Rosen N, Iny Stein T, Shmoish M, Nativ N, Bahir I, Doniger T, Krug H, Sirota-Madi A, Olender T, Golan Y, Stelzer G, Harel A, Lancet D (2010). GeneCards Version 3: the human gene integrator. Database.

[30] Szklarczyk D, Morris JH, Cook H, Kuhn M, Wyder S, Simonovic M, Santos A, Doncheva NT, Roth A, Bork P, Jensen LJ, von Mering C (2017). The STRING database in 2017: quality-controlled protein-protein association networks, made broadly accessible. Nucleic Acids Res.

[31] Sobrinho Santos EM, Almeida AC, Santos HO, Cangussu AR, Costa KS, Alves JN, Bertucci Barbosa LC, Souza Aguiar RW (2019). Mechanism of Brassica oleracea performance in bovine infectious mastitis by bioinformatic analysis. Microb Pathog.

[32] Pan Q, Zhou R, Su M, Li R. (2019). The effects of plumbagin on pancreatic cancer: a mechanistic network pharmacology approach. Med Sci Monit.

[33] Szklarczyk D, Gable AL, Lyon D, Junge A, Wyder S, Huerta-Cepas J, Simonovic M, Doncheva NT, Morris JH, Bork P, Jensen LJ, Mering CV (2019). STRING v11: protein-protein association networks with increased coverage, supporting functional discovery in genome-wide experimental datasets. Nucleic Acids Res.

[34] Szklarczyk D, Gable AL, Nastou KC, Lyon D, Kirsch R, Pyysalo S, Doncheva NT, Legeay M, Fang T, Bork P, Jensen LJ, von Mering C (2021). The STRING database in 2021: customizable protein-protein networks, and functional characterization of user-uploaded gene/measurement sets. Nucleic Acids Res.

[35] Zhou Y, Zhou B, Pache L, Chang M, Khodabakhshi AH, Tanaseichuk O, Benner C, Chanda SK (2019). Metascape provides a biologist-oriented resource for the analysis of systems-level datasets. Nat Commun.

[36] Wang R, Xu X, Yang J, Chen W, Zhao J, Wang M, Zhang Y, Yang Y, Huang W, Zhang H (2023). BPDE exposure promotes trophoblast cell pyroptosis and induces miscarriage by up-regulating lnc-HZ14/ZBP1/NLRP3 axis. J Hazard Mater.

[37] Deng M, Tong R, Bian Y, Hou G (2023). Astaxanthin attenuates cigarette smoking-induced oxidative stress and inflammation in a sirtuin 1-dependent manner. Biomed Pharmacother.

[38] Zhao J, Meng M, Zhang J, Li L, Zhu X, Zhang L, Wang C, Gao M (2019). Astaxanthin ameliorates renal interstitial fibrosis and peritubular capillary rarefaction in unilateral ureteral obstruction. Mol Med Rep.

[39] Zhou L, Xue C, Chen Z, Jiang W, He S, Zhang X (2022). c-Fos is a mechanosensor that regulates inflammatory responses and lung barrier dysfunction during ventilator-induced acute lung injury. BMC Pulm Med.

[40] Smith KM, Mrozek JD, Simonton SC, Bing DR, Meyers PA, Connett JE, Mammel MC (1997). Prolonged partial liquid ventilation using conventional and high-frequency ventilatory techniques: gas exchange and lung pathology in an animal model of respiratory distress syndrome. Crit Care Med.

[41] Livak KJ, Schmittgen TD (2001). Analysis of relative gene expression data using real-time quantitative PCR and the 2(-Delta Delta C(T)) Method. Methods.

[42] Bellani G, Laffey JG, Pham T, Fan E, Brochard L, Esteban A, Gattinoni L, van Haren F, Larsson A, McAuley DF, Ranieri M, Rubenfeld G, Thompson BT, Wrigge H, Slutsky AS, Pesenti A (2016). Epidemiology, patterns of care, and mortality for patients with acute respiratory distress syndrome in intensive care units in 50 countries. Jama.

[43] Algera AG, Pisani L, Chaves RCF, Amorim TC, Cherpanath T, Determann R, Dongelmans DA, Paulus F, Tuinman PR, Pelosi P, Gama de Abreu M, Schultz MJ, Serpa A (2018). Effects of peep on lung injury, pulmonary function, systemic circulation and mortality in animals with uninjured lungs-a systematic review. Ann Transl Med.

[44] Moraes L, Silva PL, Thompson A, Santos CL, Santos RS, Fernandes MVS, Morales MM, Martins V, Capelozzi VL, Abreu MG, Pelosi P, Rocco PRM (2018). Impact of different tidal volume levels at low mechanical power on ventilator-induced lung injury in rats. Front Physiol.

[45] Pham T, Brochard LJ, Slutsky AS (2017). Mechanical ventilation: state of the art. Mayo Clin Proc.

[46] Zhou X, Cao Q, Orfila C, Zhao J, Zhang L (2021). Systematic review and meta-analysis on the effects of astaxanthin on human skin ageing. Nutrients.

[47] Balendra V, Singh SK (2021). Therapeutic potential of astaxanthin and superoxide dismutase in Alzheimer’s disease. Open Biol.

[48] Catanzaro E, Bishayee A, Fimognari C (2020). On a beam of light: photoprotective activities of the marine carotenoids astaxanthin and fucoxanthin in suppression of inflammation and cancer. Mar Drugs.

[49] Hong M, Nie Z, Chen Z, Bao B (2024). Astaxanthin attenuates diabetic kidney injury through upregulation of autophagy in podocytes and pathological crosstalk with mesangial cells. Ren Fail.

[50] Lee H, Lim JW, Kim H (2020). Effect of astaxanthin on activation of autophagy and inhibition of apoptosis in helicobacter pylori-infected gastric epithelial cell line AGS. Nutrients.

[51] Yan T, Ding F, Zhang Y, Wang Y, Zhang Y, Zhu F, Zhang G, Zheng X, Jia G, Zhou F, Zhao Y, Zhao Y (2024). Astaxanthin inhibits H(2)O(2)-induced excessive mitophagy and apoptosis in SH-SY5Y cells by regulation of Akt/mTOR activation. Mar Drugs.

[52] Lim SR, Kim DW, Sung J, Kim TH, Choi CH, Lee SJ (2021). Astaxanthin inhibits autophagic cell death induced by bisphenol A in human dermal fibroblasts. Antioxidants (Basel).

